# Stimulator of interferon genes (STING) activation exacerbates experimental colitis in mice

**DOI:** 10.1038/s41598-019-50656-5

**Published:** 2019-10-03

**Authors:** Gary R. Martin, Charlene M. Blomquist, Kimiora L. Henare, Frank R. Jirik

**Affiliations:** 1 Department of Biochemistry & Molecular Biology and The McCaig Institute for Bone & Joint Health, University of Calgary, Calgary, Alberta Canada; 2 Auckland Cancer Society Research Centre, The University of Auckland, Auckland, New Zealand

**Keywords:** Ulcerative colitis, Acute inflammation

## Abstract

Detection of cytoplasmic DNA by the host’s innate immune system is essential for microbial and endogenous pathogen recognition. In mammalian cells, an important sensor is the stimulator of interferon genes (STING) protein, which upon activation by bacterially-derived cyclic dinucleotides (cDNs) or cytosolic dsDNA (dsDNA), triggers type I interferons and pro-inflammatory cytokine production. Given the abundance of bacterially-derived cDNs in the gut, we determined whether STING deletion, or stimulation, acts to modulate the severity of intestinal inflammation in the dextran sodium sulphate (DSS) model of colitis. DSS was administered to *Tmem173*^*gt*^ (STING-mutant) mice and to wild-type mice co-treated with DSS and a STING agonist. Colitis severity was markedly reduced in the DSS-treated *Tmem173*^*gt*^ mice and greatly exacerbated in wild-type mice co-treated with the STING agonist. STING expression levels were also assessed in colonic tissues, murine bone marrow derived macrophages (BMDMs), and human THP-1 cells. M1 and M2 polarized THP-1 and murine BMDMs were also stimulated with STING agonists and ligands to assess their responses. STING expression was increased in both murine and human M1 polarized macrophages and a STING agonist repolarized M2 macrophages towards an M1-like subtype. Our results suggest that STING is involved in the host’s response to acutely-induced colitis.

## Introduction

Bacterial-derived cyclic dinucleotides (cDNs) are ubiquitous products of bacterial metabolism that are responsible for regulating diverse processes, both within gram-negative and gram-positive bacteria, that include motility, virulence, stress survival, biofilm production, differentiation and metabolism^1,2^. The primary target for bacterially-derived cDNs as well as the ‘non-canonical’ cDNs generated by cyclic GMP-AMP synthase (cGAS), a key eukaryotic cell sensor for cytosolic dsDNA^3–5^, is the ER membrane-resident protein, STING^6–10^. Downstream effects of STING activation include NFκB, IRF3 and STAT6 activation, which leads to the production of Type I interferons and pro-inflammatory cytokine production and M2-to-M1 macrophage repolarization^2,11^. Activation of STING by cGAS-generated cDN, cyclic GMP-AMP (cGAMP), is pivotal to the hosts defense against viruses^12^ and during circumstances whereby dsDNA fragments derived from bacteria, viruses, or host cells themselves, are present in the cytosol^13^. For example, it was reported that STING exerted a protective role against inflammation-induced colorectal cancer^14^ and also that mice lacking STING had reduced resistance towards mutagen-induced, inflammation-driven, epithelial cancer^15^. cDNs are now considered members of the PAMP family as they function as ligands for the pattern recognition receptor STING, thus stimulating innate immunity^2^. Given the abundance of cDNs within the lumen of the lower GI tract, STING activation by cDNs, and possibly cytosolic-derived DNA, it is plausible that STING plays a role in regulating the inflammatory response of the host during dysbiosis as well as other conditions involved in both the onset and sustainment of colitis.

Humans express STING^8,16^, however, the role of STING in the human gastrointestinal tract remains unknown. In other species, for example in mice, the role of STING in the gut appears to be discordant as some report that STING deficient mice are susceptible to experimentally-induced inflammation and that the molecule controls the degree of inflammation^14,17^, whereas others have shown that STING-deficient mice are rescued from spontaneous colitis^18^.

In contrast with its role in host defense^12–14^, it was observed that sepsis severity is reduced in STING-deficient mice relative to wild-type mice in the cecal ligation and puncture model of sepsis^19–21^. In addition, acute or chronic carbon tetrachloride administration to wild-type mice resulted in early endoplasmic reticulum stress, type I interferon production, hepatocyte apoptosis and liver fibrosis, whereas hepatocyte death and fibrosis in similarly treated mice lacking STING was prevented^22^. There is also evidence that STING deficiency reduces pro-inflammatory cytokine production and arthritis scores in a murine model of cytosolic self-DNA-mediated autoimmunity^23^. In addition, STING gain-of-function mutations lead to an auto-inflammatory disease, typically manifested in infancy, that exhibits a severe acral vasculopathy and lung inflammation, amongst other features^24^.

Thus, STING appears able to serve a protective function, involved in host defense against viruses and bacteria and injury repair, or alternatively, as a potentially pathogenic pro-inflammatory mediator depending on the nature and severity of the insult. While there is much evidence that excessive STING activation plays a role in triggering various disease states, including autoimmunity and autoinflammatory disease^19,23,24^, the role of STING as a regulator of the response of the host to colitis remains largely undefined. Hence, we examined the role of STING activation and/or its deficiency in the DSS-induced model of experimental colitis. While this model does not replicate all aspects of human colitis, it does share some similarities including epithelial barrier dysfunction, cytokine dysregulation, and mucosal pathology and thus has proven very useful when examining the functions of genes and mediators involved in the intestinal immune response to injury and bacterial invasion of colonic tissue^25^.

While it was possible that treatments with a STING agonist could have improved the inflammation associated with DSS-colitis, we hypothesized that it would have pro-inflammatory effects. Herein we identified that DSS-treated normal mice that were co-treated with a STING agonist developed a dramatic worsening of colitis whereas similarly treated mice that lacked the STING gene had a reduction in disease severity. We also observed a decreased expression of the macrophage activation marker Iba-1 in response to DSS in these STING-KO mice and that STING activation of murine BMDMs by a STING agonist or STING ligands re-polarized M2 macrophages towards a pro-inflammatory M1-like subtype. Lastly, DSS-induced colitis, as well as pharmacological activation of STING *in vitro*, were found to increase STING protein expression.

## Results

### Agonist-induced STING activation worsened, whereas STING deficiency reduced weight loss and colonic shortening in response to DSS

**Figure 1:**
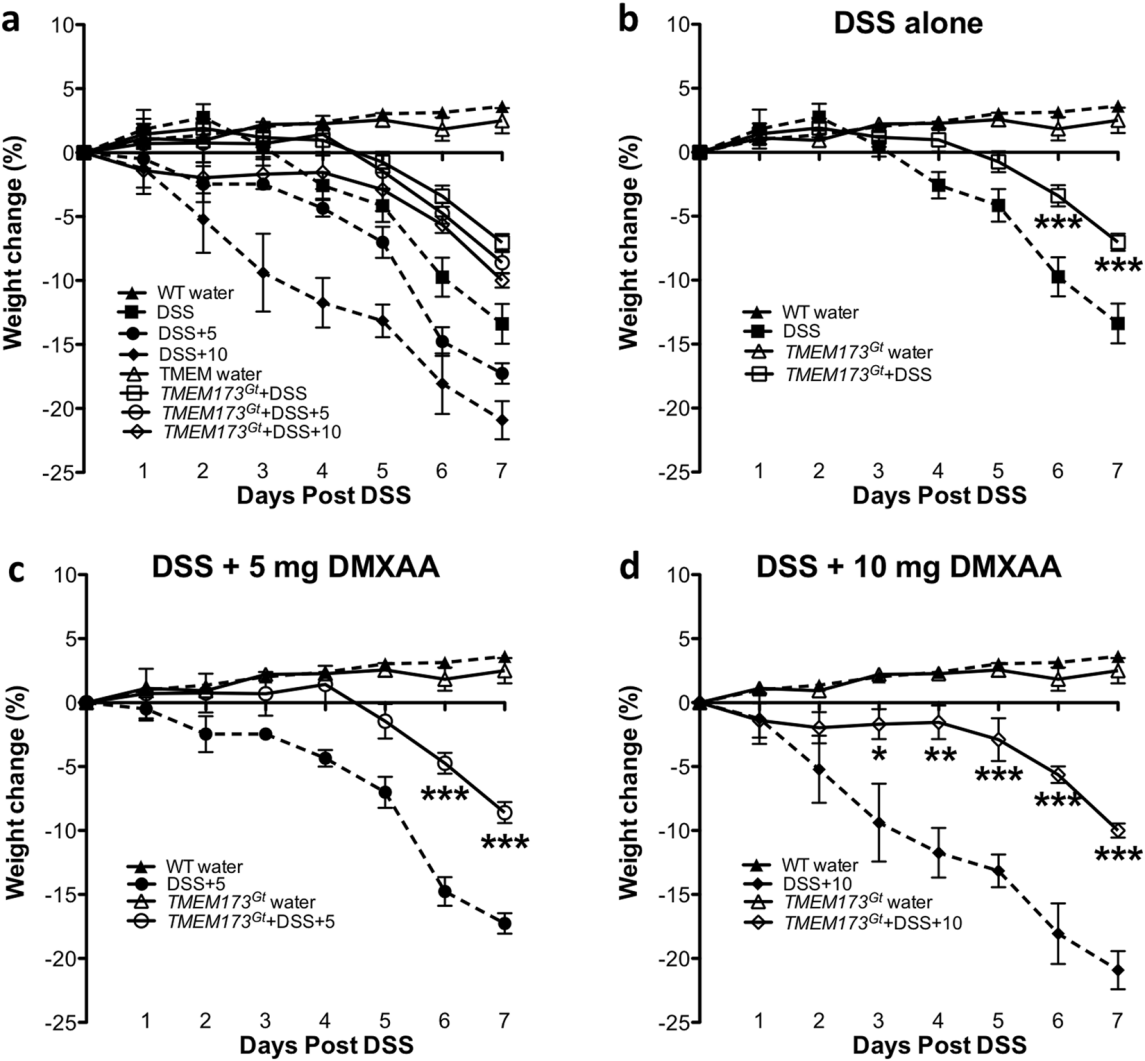
Weight losses associated with DSS-induced colitis in *Tmem17*^*Gt*^ and wild-type mice. (**a**) Weight losses were exacerbated in the DSS mice that were co-treated with the murine STING agonist, DMXAA, and attenuated in the *Tmem173*^*Gt*^ group of mice. To examine the effects of a given treatment regime on weight change, we isolated groups and compared the effects of (**b**) DSS-induced colitis either alone or following co-treatments with (**c**) 5 mg/kg or (**d**)10 mg/kg of DMXAA (i.p.). Statistical significance between data sets was assessed by one-way ANOVA followed by Tukey’s multiple comparisons post-hoc test between all groups. Values are means ± SEM, n ≥ 8 mice per group with differences denoted by *P < 0.05,**P < 0.01, ***P < 0.001 relative to DSS-treated WT.

**Figure 2:**
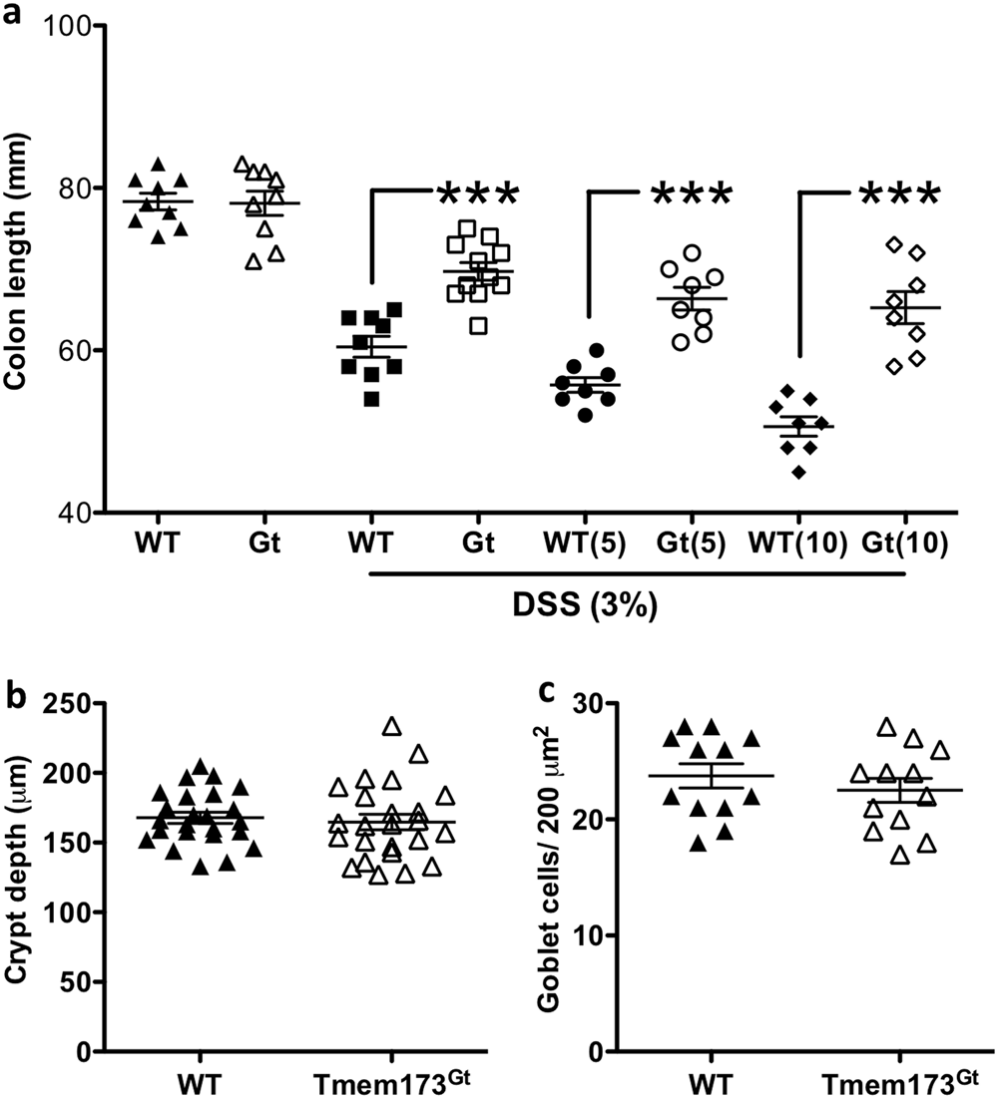
Colonic shortening. (**a**) DSS treatment induced colonic shortening in all treatment groups, but relative to DSS-treated WT mice, was significantly greater in the WT DSS mice that had also received DMXAA. In contrast, this effect was significantly reduced in both the *Tmem173*^*gt*^ and the *Tmem173*^*gt*^ mice co-treated with the STING agonist. Black-filled dots are for WT, or WT + 5 or + 10 mg/kg DMXAA; white-clear dots are for the *Tmem173*^*gt*^ receiving similar treatments. Statistical significance between data sets was assessed by one-way ANOVA followed by Tukey’s multiple comparisons post-hoc test between all groups. Values are means ± SEM, n ≥ 8 mice per group, ***P < 0.001 longer relative to DSS-treated WT. Analysis of (**b**) crypt depth or (**c**) goblet cell numbers in the colons of the untreated WT and *TMEM173*^Gt^ mice. To assess crypt depth, 4 random locations from 6 untreated WT or *TMEM173*^Gt^ mice (n = 24 total) measurements were plotted. Upon statistical comparison, there were no significant differences in crypt height or goblet cell numbers when sections from untreated WT and *Tmem173*^*gt*^ mice were compared. Goblet cell counts represent the number of goblet cells per 200 um^2^ area, n = 12, Student’s T-test was used to determine possible differences between data sets.

Although all mice that received DSS experienced drops in body weight relative to tap water-treated controls, weight loss was aggravated in the WT mice treated with the murine STING agonist DMXAA^26^, but was significantly reduced in the *Tmem173*^*gt*^ mice (Fig. 1a–d). Weight changes in the non-DSS treated WT mice that received 5 mg/kg/day of DMXAA alone via i.p. injection were not significantly different when compared to tap water-treated controls (data not shown). As with weight loss, colonic shortening occurred in all DSS-treated mice, however, the degree of colonic shortening in the DSS-treated WT mice that were co-treated with DMXAA was significantly greater (Fig. 2a). In contrast, DSS-induced colonic shortening in *Tmem173*^*gt*^ mice, either with or without DMXAA treatment, was considerably less. This indicated that the exacerbation of DSS-induced colitis was being driven by STING activation.

### Agonist-induced STING activation exacerbated DSS-induced colonic damage and inflammation

**Figure 3:**
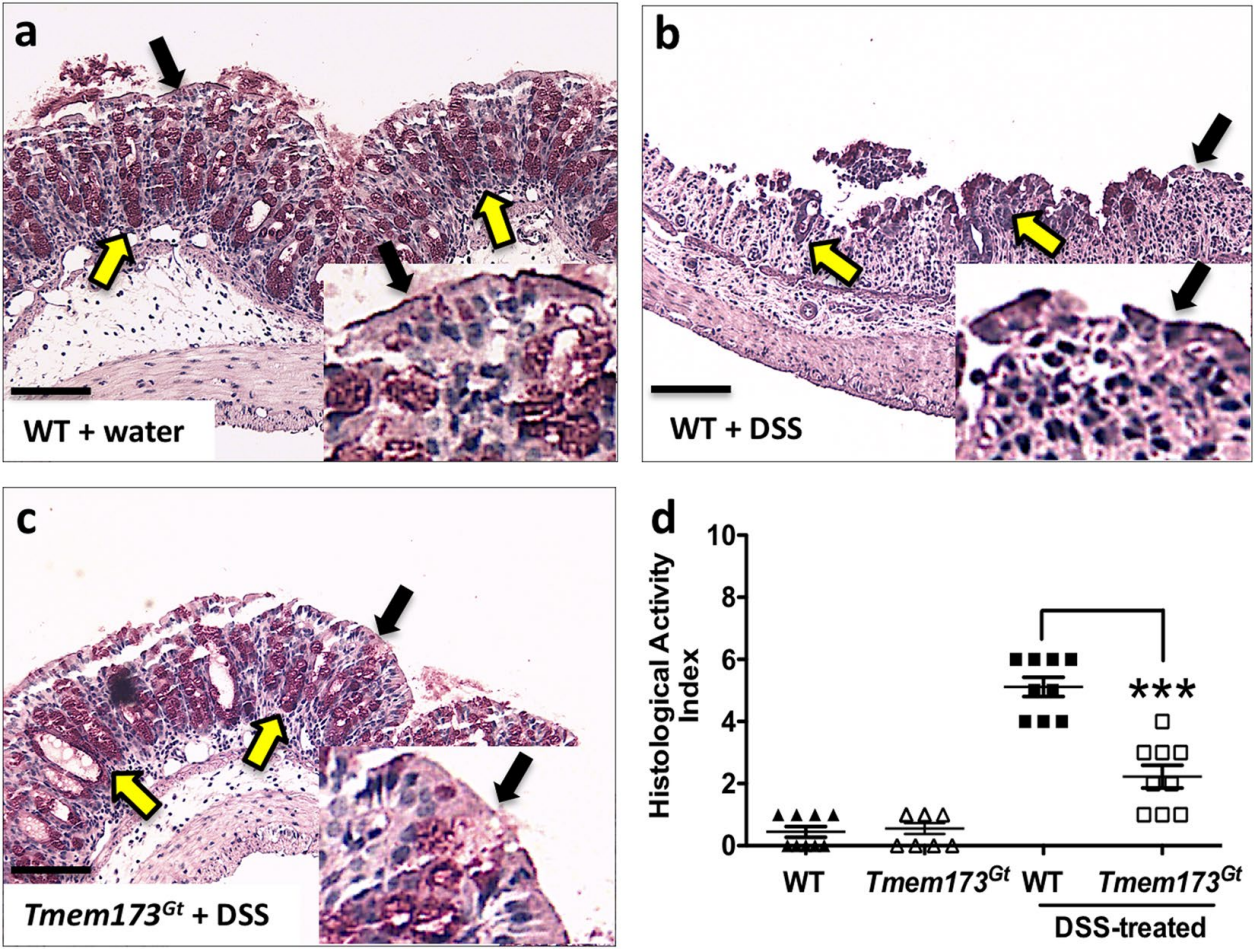
STING-deficient mice demonstrate attenuation of DSS-induced acute colitis. Representative histological sections harvested from (**a**) WT, (**b**) DSS-treated WT and (**c**) DSS-treated *Tmem173*^*gt*^ mice. After 7 days of DSS treatment, crypt loss, as well as infiltration and hypertrophy, was reduced in the DSS-treated *Tmem173*^*gt*^ mice when compared to WT mice. In addition, epithelial brush border (black arrowheads) and goblet cell (yellow arrowheads) losses were reduced in the colonic tissues of DSS-treated *Tmem173*^*gt*^ mice when compared to similarly treated WT controls. (**d**) Although the HAI was elevated in all DSS-treated mice when compared to non-treated WT mice, colonic damage in the DSS-treated *Tmem173*^*gt*^ mice was markedly reduced. PAS stain. Sections imaged using a 20x objective. Statistical significance was assessed by one-way ANOVA followed by Tukey’s multiple comparisons post-hoc test between all groups. Values represent means ± SEM, n ≥ 8 mice per group, ***P < 0.001 relative to DSS-treated WT.

**Figure 4:**
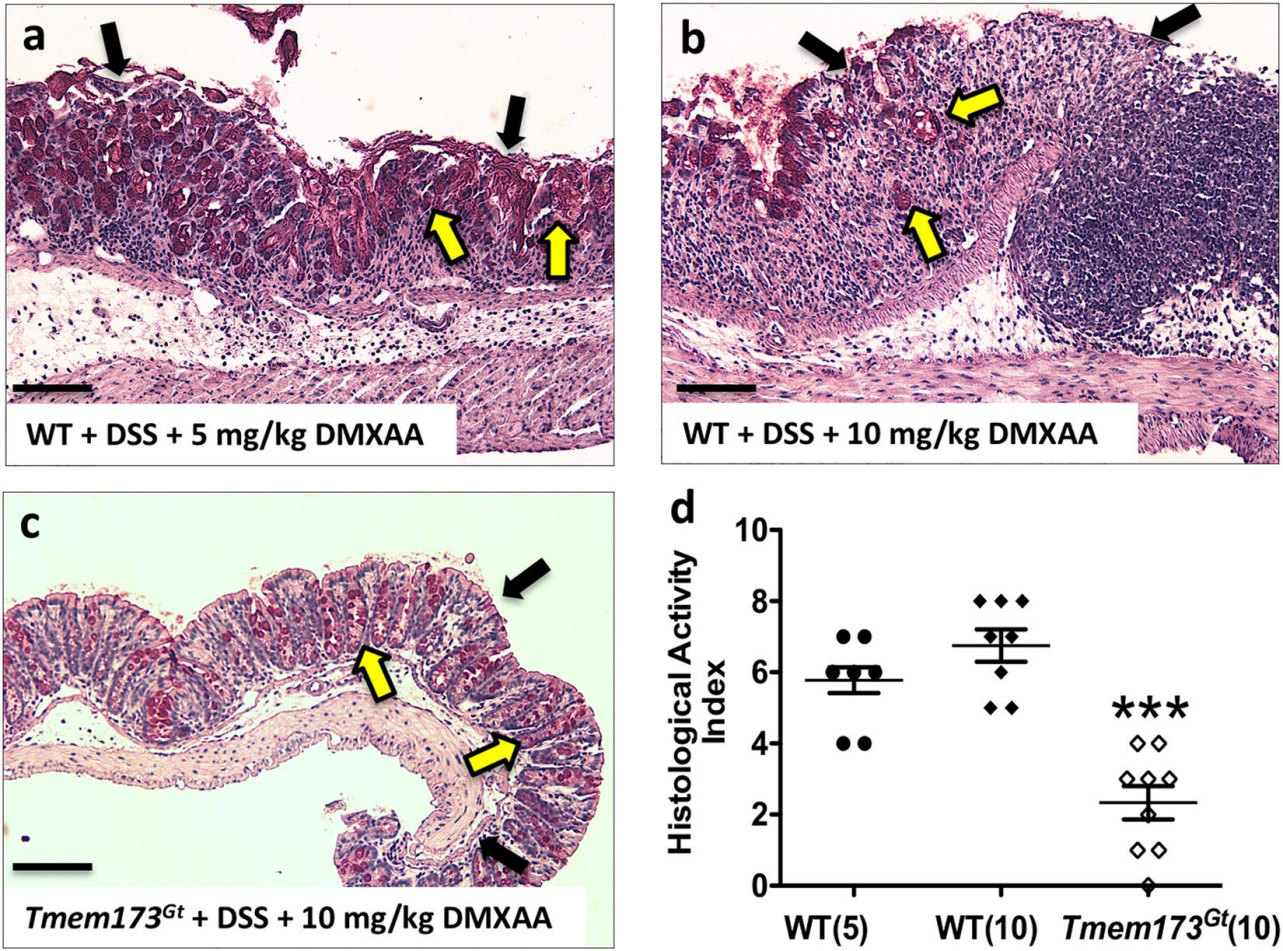
Administration of a STING agonist exacerbates DSS-induced colitis. Sections were harvested from DSS-treated WT mice that were co-treated with (**a**) 5 mg/kg/day of DMXAA or from (**b**) WT or (**c**) *Tmem173*^*gt*^ mice co-treated with 10 mg/kg/day of DMXAA every other day. (**d**) Inflammation-as assessed using the histological activity index (HAI), an index that provides a damage score-in the WT mice that were administered DMXAA was more severe than that of DMXAA-treated *Tmem173*^*gt*^ mice. Note the loss of the epithelial brush border (black arrowheads) and goblet cells (yellow arrowheads). Colonic damage in the *Tmem173*^*gt*^ mice co-treated with DMXAA was markedly reduced when compared to both WT groups. Sections were harvested on day 7. PAS stain. All sections imaged using a 20x objective; black bars at the bottom left of the panels represent 200 μM. Statistical significance was assessed by one-way ANOVA followed by Tukey’s multiple comparisons post-hoc test between all groups. Values for (D) are the means ± SEM, n ≥ 7 mice per group, ***P < 0.001 relative to both the WT(5) and WT(10) groups of mice.

We next examined histological sections using a standardized, commonly employed method for assessing intestinal pathology. This incorporates both epithelial damage and the degree of inflammatory infiltrate to assign a score for colonic damage, that is, the histological activity index (HAI). Histologically, unlike the report by Canesso *et al*.^17^, we did not observe any significant colonic mucosal abnormalities (eg. crypt depth, Fig. 2b or goblet cell numbers, Fig. 2c) when sections from untreated WT and *Tmem173*^*gt*^ mice were compared. The HAI was elevated in the DSS-treated mice groups when compared to respective tap water-treated groups of mice (Fig. 3a–d), however, the degree of colonic damage, hypertrophy, infiltration, and muscle thickening with edema in the DSS-treated wild-type mice that had also been treated with the STING agonist was significantly worse (Fig. 4a–c). In addition, no exacerbation of DSS-induced colitis was detected in *Tmem173*^*gt*^ mice that had received daily injections of DMXAA (Fig. 4c). Furthermore, epithelial damage and crypt cell losses in DSS-treated *Tmem173*^*gt*^ mice were markedly reduced (Fig. 4d). Brush border membrane and goblet cell losses were accentuated in DSS-treated WT mice that were also treated with DMXAA (Fig. 4d). Consistent with STING being the principal target of DMXAA in mice, colonic damage was attenuated in the DMXAA- plus DSS-treated *Tmem173*^*gt*^ mice. In addition, there were no overt signs of hemorrhagic necrosis observed in the colons of these mice.

### Colitis-induced expression of the macrophage activation marker Iba-1 was reduced in *Tmem173*^*gt*^ mice

**Figure 5:**
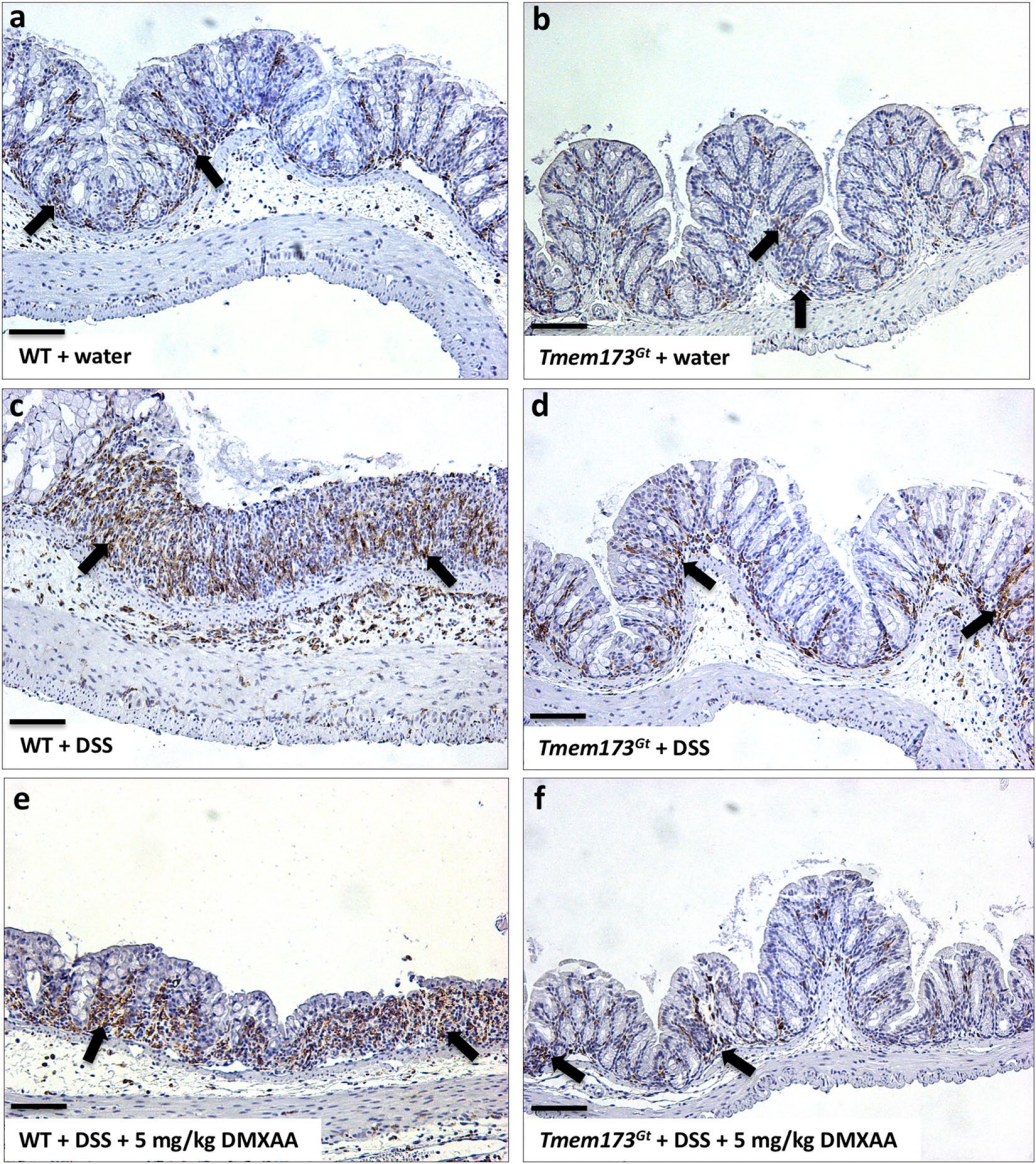
DSS-induced colitis increased the numbers of activated macrophages in WT mice. To assess macrophage activation, paraffin sections from the various treatment groups were stained with Iba-1, a protein upregulated during activation of macrophages and possibly neutrophils (black arrows). Representative sections harvested from vehicle-treated (**a**) WT or (**b**) *Tmem173*^*gt*^ mice, or from DSS-treated (**c**) WT or (**d**) *Tmem173*^*gt*^ mice or from DSS treated (**e**) WT or (**f**) *Tmem173*^*gt*^ mice that were co-treated with DMXAA (5 mg/kg). Although there were no discernable differences in macrophage activation between the untreated WT and *Tmem173*^*gt*^ groups of mice, Iba-1 staining of colonic tissues from the DSS-treated WT and DSS-treated WT mice co-treated with DMXAA looked to be increased when compared with similarly-treated *Tmem173*^*gt*^ mice. All sections were harvested on day 7 from mice treated with 3% DSS. Anti-Iba-1 immuno-histochemical stain. Sections imaged using a 20x objective.

Since the presence or absence of STING protein regulated the severity of DSS-induced colitis, we investigated whether inflammation might regulate the numbers of activated macrophages. We thus stained paraffin sections of DSS-exposed colons with Iba-1, a protein expressed in macrophages, microglia (and possibly in neutrophils), that is upregulated during activation, to assess whether STING activation or absence would influence Iba-1 expression in these colons. There were no overt observable differences in the expression of Iba-1 when tap water-treated WT and *Tmem173*^*gt*^ tissues were compared (Fig. 5a,b). Though no formal measurements were utilized, colonic inflammation appears to have increased the number of activated macrophages (as per visual assessment of Iba-1 staining) in all DSS-treated groups (Fig. 5c–f). However, this increase in Iba-1 staining was markedly attenuated in the *Tmem173*^*gt*^ mice as compared to WT mice that received similar treatments (Fig. 5c–f).

### STING protein expression is increased by colitis and M1 polarization of murine or human THP-1-derived macrophages

**Figure 6:**
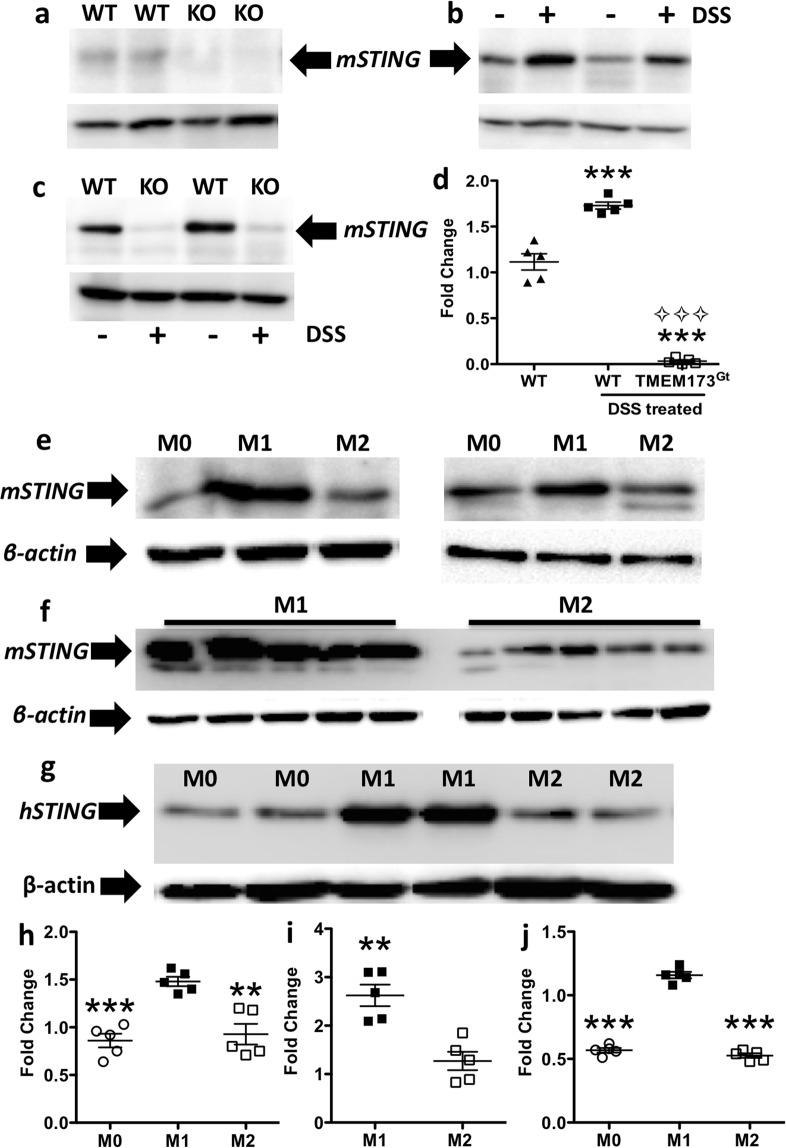
Colitis and M1 polarization of murine and human macrophages increased STING protein expression. (**a**) A representative blot of STING expression from the colonic lysates of two untreated WT or *Tmem173*^*gt*^ (KO) mice. (**b–d**) DSS-induced colitis increased STING protein expression in WT mice (+ = DSS treated) relative to that observed in both tap water treated control mice (− = tap water only) and STING deficient mice. One-way ANOVA followed by Tukey’s multiple comparisons post-hoc test between all groups was used. Values for (**d**) are means ± SEM, n = 5 mice per group, ***P < 0.001 relative to both the WT and *Tmem173*^*gt*^ groups and ^⟡⟡⟡^P < 0.001 relative to DSS-treated WT mice. (**c,h**) Representative blots and a histogram from two mice to show that STING protein expression in murine BMDM lysates was increased in M1 polarized macrophages when compared to M2 polarized or M0 macrophages. (**d**) To confirm the previous finding in (**c**), BMDMs from 5 mice were polarized into M1 or M2 macrophages. **(i**) Again, there was a significant increase in mSTING expression in the M1 polarized macrophages relative to the expression levels found in the M2 cells. (**g,j**) This also occurred in human THP-1 monocytic cells as M1 polarization markedly increased STING protein expression. Immunoblots of polarized murine BMDMs: M0, untreated; M1, 50 ng/ml of LPS and IFNγ; M2, 40 ng/ml IL-4 for 48 hrs. 50 μg of protein/lane was loaded, and β-actin was used as the loading control for all immunoblot analyses. For (**h,j**) one-way ANOVA followed by Tukey’s multiple comparisons post-hoc test between all groups was used, n = 5, **P < 0.01 or ***P < 0.001 relative to M0 or M2; for (**i**), a Student’s T-test was applied with **P < 0.01 vs. M2.

STING protein expression was detected in colonic tissue lysates obtained from control WT mice, but not from *Tmem173*^*gt*^ mice (Fig. 6a). We also observed that STING protein expression in the colonic lysates from the WT mice that had been administered DSS was significantly increased relative to that found in the WT littermates that received tap water only (Fig. 6b,d). In addition, STING protein expression levels were only detected in the colonic lysates obtained from DSS-treated WT mice, but not from *Tmem173*^*gt*^ mice (Fig. 6c). Since whole colonic tissue sections were analyzed, it was not possible to discern which cell types were contributing to the STING protein levels. However, since M1 polarized macrophages have a key role in the pathogenesis of DSS-induced colitis^27,28^ we investigated whether these cells exhibited increased STING protein levels in response to M1-polarizing stimuli. Indeed, STING protein levels were dramatically increased under M1 polarizing conditions (Fig. 6e,f,h,i). In contrast, both M0 (no polarizing factors) or M2 polarized macrophages demonstrated much lower STING levels (Fig. 6e,f,h). Similarly, STING levels were dramatically increased in PMA-differentiated human THP-1-derived macrophages in response to M1 polarizing stimuli, while M2 polarization led to no significant change in STING expression levels (Fig. 6g,j). Thus, M1 polarization of human or murine cells with LPS and IFNγ was associated with a substantial increase in STING protein expression, a finding that might predict an increased sensitivity of M1 polarized cells to either bacterially- or endogenously-derived cDNs.

### Treatment of BMDMs with a STING agonist induces a pro-inflammatory subtype

We next sought to explain why DSS colitis was exacerbated by co-treatment with a STING agonist. While it has been shown that M1 polarization can exacerbate DSS-induced colitis, the associated inflammation was attenuated by genetic manipulations favouring differentiation of the M2 phenotype^27,29,30^. Previously, we showed that the activation of the STING pathway with a STING agonist was able to induce the repolarization of murine M2 macrophages, both *in vitro* and *in vivo*, towards an M1-like phenotype^11^ which possibly explains the adverse effect of DMXAA on colitis severity.

**Figure 7:**
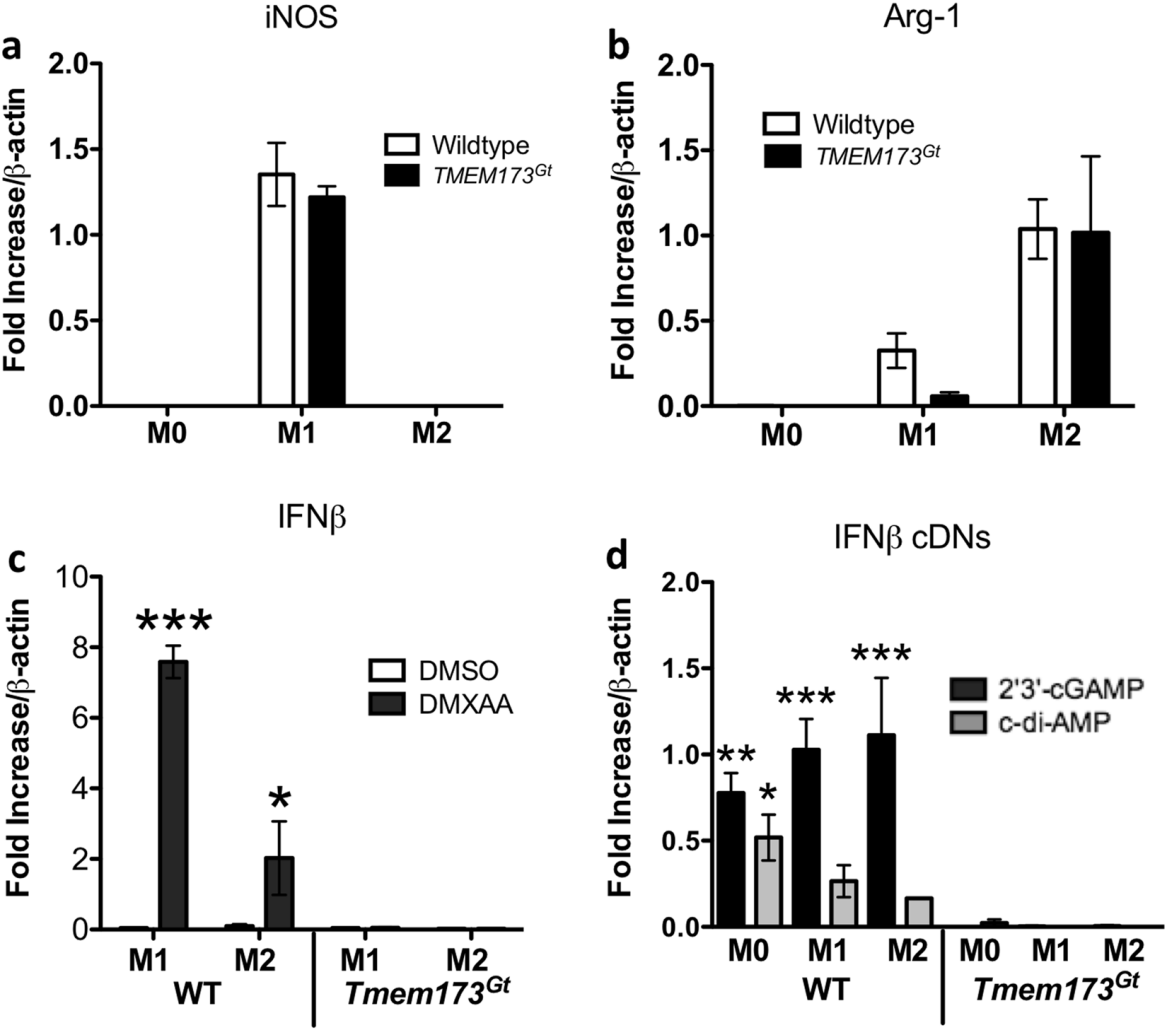
STING agonist induction of IFN-β. Bone marrow-derived macrophages (BMDMs) derived from the femurs of WT or *Tmem173*^*gt*^ mice were polarized *in vitro* into M1 or M2 subtypes. **(a,b)** Using iNOS/NOS2 mRNA induction as a marker for M1 cells, and Arg-1 mRNA induction as a marker for the M2 subtype, we observed no differences in the expression of either marker when these cells from the WT and *Tmem173*^*gt*^ were compared. **(c)** Increased IFN-β expression or secretion in response to STING agonists (or cytoplasmic DNA exposure) is a commonly used marker to show that the STING pathway has been activated. In this regard, when M1 or M2 polarized cells from the WT or *Tmem17*^*Gt*^ were incubated for 6-hr with DMXAA, it was observed that IFN-β mRNA expression was only induced in the WT macrophages. **(d)** Similarly, when the 3 subtypes of macrophages were exposed to the non-canonical ligand 2′3′-cGAMP or the bacterial second messenger c-di-AMP, IFN-β expression was increased solely in the WT macrophage polarized subsets. Significance was assessed by one-way ANOVA followed by Tukey’s multiple comparisons post-hoc test between all groups. Values represent the means ± SEM of the fold changes in the respective mRNAs; *P < 0.05, **P < 0.01, or ***P < 0.001 vs. the corresponding *Tmem17*^*Gt*^ group (eg. WT M1, DMXAA treated vs. *Tmem17*^*Gt*^ M1, DMXAA treated). For macrophage polarization: M0, untreated; M1, 50 ng/ml of LPS and IFNγ; M2, 40 ng/ml IL-4 for 48 hrs. STING agonists: DMXAA 20 μg/ml; c-di-AMP 20 μg/ml plus lipofectamine; 2′3′-cGAMP 20 μg/ml plus lipofectamine for 6 hrs. Data was normalized to β-actin mRNA and experimental transcripts expressed as the relative fold change in each mRNA selected.

Based on expression of the murine M1 marker iNOS, and M2 marker, Arg-1, macrophages from *Tmem173*^*gt*^ mice polarized *in vitro* into both the M1 and M2 phenotypes similar to WT macrophages (Fig. 7a,b). Thus, cells lacking STING were able to respond to M1 (IFNγ plus LPS) or M2 (IL-4) polarizing stimuli. In contrast, *Tmem173*^*gt*^ macrophages treated with a STING agonist were hypo-responsive (eg. failed to generate IFNβ following DMXAA treatment) when compared to that secreted by WT macrophages (Fig. 7c). Similar results were observed using the natural STING ligands, bacterial c-di-AMP or mammalian 2′3′-cGAMP, as the production of the cytokine was only observed in the WT macrophages (Fig. 7d).

**Figure 8:**
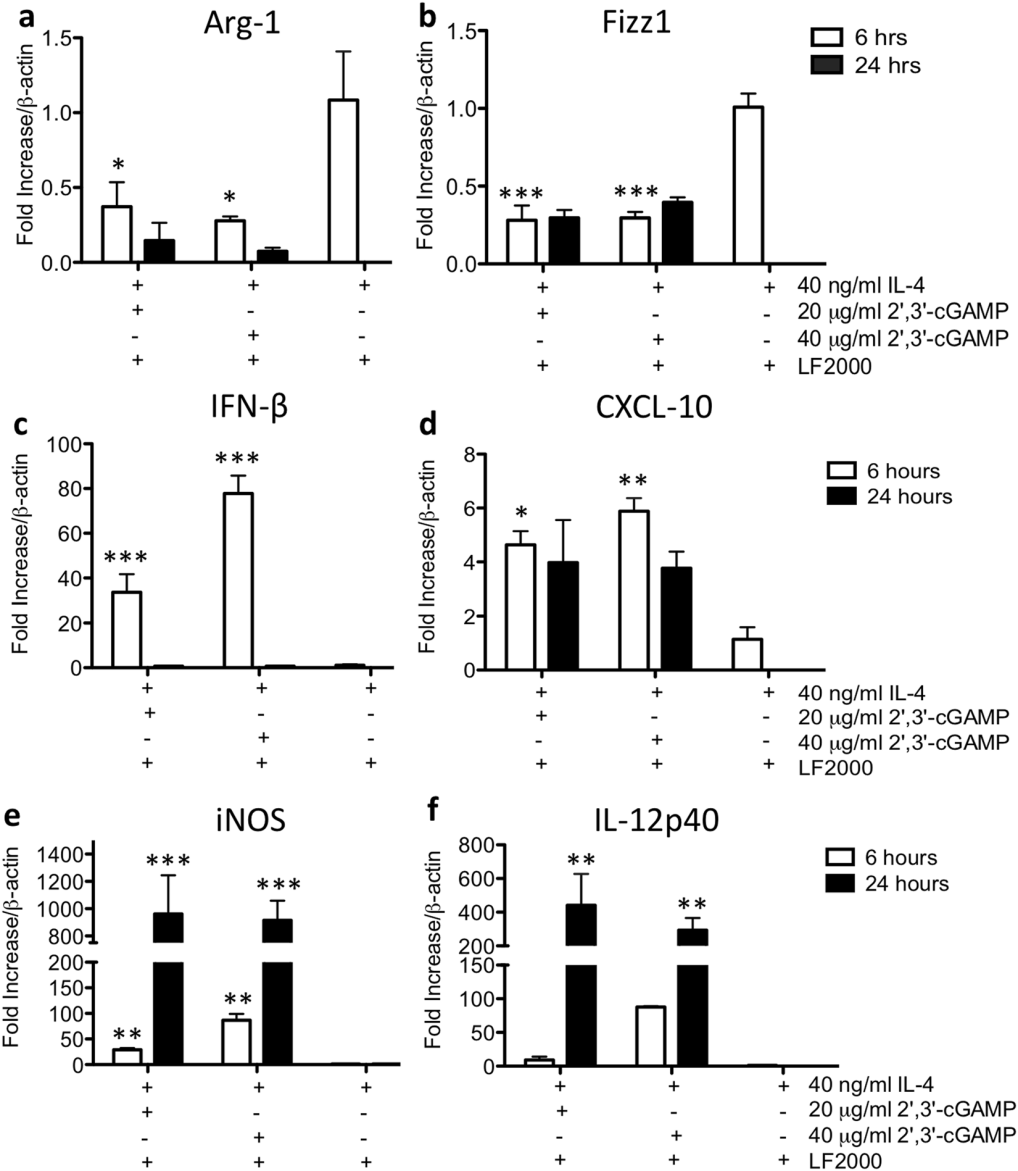
cDN-induced repolarization of M2 polarized macrophages towards the M1 pro-inflammatory subtype. To establish M2 subtype of macrophages, cells were pre-treated with 40 ng/ml IL-4 for 48 hrs prior to the addition of cDNs. (**a**,**b)** A 6-hr treatment with either 2′3′-cGAMP or c-di-AMP significantly decreased Arg-1 and Fizz1 mRNA expression as compared to the IL-4-alone treated cells. (**c**–**f**) Moreover, IFN-β, CXCL-10, iNOS and IL-12p40 mRNAs were also increased at either the 6 hr or the 24 hr timepoint post-cDN exposure. All cDN treatments were carried out using lipofectamine permeabilization of the cultured cells. Significance was assessed by one-way ANOVA followed by Tukey’s multiple comparisons post-hoc test between all groups. Values represent the means ± SEM of the fold changes in the respective mRNAs; *P < 0.05, **P < 0.01, ***P < 0.001 vs. the control groups treated with IL-4 alone. Data was normalized to β-actin mRNA and experimental transcripts expressed as the relative fold change in each mRNA tested.

Importantly, and in keeping with our previous study on 129/SvJ macrophages^11^, WT M2-polarized C57BL/6 macrophages treated with 2′3′-cGAMP or c-di-AMP also underwent M1-like repolarization. This was demonstrated by a decrease in the M2 macrophage markers, Arg-1 and Fizz1 (Fig. 8a,b), and an increase in the expression levels of potential markers of M1-like activity (including IFNβ, CXCL-10, iNOS and IL-12p40 transcripts) in the M2 population (Fig. 8c,d). While effective delivery of these two cDNs into macrophages required lipofectamine, we previously showed that this treatment does not activate or polarize these cells^11^. Thus, one possible explanation as to why the co-administration of DSS and a STING agonist led to a dramatic worsening of colitis, is that the STING agonists increased pro-inflammatory activities in the M1 cells and repolarized M2 cells towards an M1-like phenotype.

## Discussion

To determine if STING signalling was involved in the host response to colonic inflammation, we examined, in the context of an experimental mouse model of colitis, whether STING activation, or conversely, STING-deficiency, would affect disease severity. While it was plausible that treatments with a STING agonist could have improved the inflammation associated with DSS-induced colitis-based upon results in which its deficiency was observed to exacerbate inflammation^14,17^-we hypothesized that it would have pro-inflammatory effects. Indeed, we found that the DSS-treated WT mice that were co-treated with the murine STING agonist DMXAA, developed a dramatic worsening of colitis. In contrast, similarly treated*Tmem173*^*gt*^ mice had reduced disease severity as well as decreased expression of the macrophage/microglia activation marker, Iba-1. We also found that STING activation of murine BMDMs by DMXAA or STING ligands (including the bacterial second messenger c-di-AMP and the non-canonical ligand 2′3′-cGAMP), re-polarized M2 macrophages towards a pro-inflammatory M1-like subtype. Of note, this effect was greatly dampened in the *Tmem173*^*gt*^ macrophages. In addition, DSS-induced colitis, as well as pharmacological activation of STING *in vitro*, increased STING protein expression levels. Collectively, these findings suggest that STING is capable of modulating inflammatory processes within the distal intestinal tract.

We found that the expression of Iba-1 in the intestinal macrophages of mice was significantly influenced by the presence (or absence) of STING and on STING activation following treatments with a murine STING agonist. Previous studies have shown that expression of Iba-1 is increased in the colonic tissues of mice in a model of trinitrobenzene sulfonic acid-induced acute colitis as well as in the acute rejection of cardiac allografts^31,32^. Furthermore, in a study of 47 IBD patients in various stages of disease (e.g. active vs. remission), it was reported that colonic Iba-1 expression was higher in the patients with active disease^33^. There are reports that Iba-1 expression was increased following addition of the pro-inflammatory cytokines IL-1β and TNF-α in a rat macrophage cell line as well as in mice with bleomycin-induced acute lung injury^34,35^. In our study, colitis appears to have increased Iba-1 in all DSS-treated groups, however, this was markedly reduced in the *Tmem173*^*gt*^ mice when compared to WT mice that received similar treatments. This finding is of significance as it suggests that STING activation could be involved in the activation of colonic macrophages during the acute phase of colitis.

Previously, we reported that M2 polarized murine macrophages treated with 2′3′-cGAMP or c-di-AMP, repolarized into a M1-pro-inflammatory subtype which suggests that the STING pathway has a role in re-educating M2 macrophages towards a more pro-inflammatory phenotype^11^. In the current study, we found that macrophages derived from *Tmem173*^*gt*^ mice polarized *in vitro* into both M1 and M2 phenotypes similar to that found in WT mice. This indicated that loss of STING did not affect polarization in response to IFN-γ and LPS or IL-4. However, and consistent with the loss of activation events dependent on the STING pathway, the *Tmem173*^*gt*^ macrophages treated with either DMXAA or various STING ligands did not respond to the same extent as the WT macrophages (eg. loss of the M2 markers Arg-1 and Fizz1 and increased expression of the M1 markers CXCL-10, iNOS and IL-12p40) and lacked IFN-β induction. This function of STING activation may have accounted for the dramatic worsening of DSS colitis we observed following DMXAA administration as the inhibition of M2 polarization has shown to worsen colitis, whereas the promotion of M2 (or inhibition of M1) polarization attenuates inflammation^28,36,37^.

We also investigated whether *in vitro* polarization of macrophages might have an effect on STING protein expression within M0, M1 or M2 polarized murine and human macrophages. We observed that STING protein levels were greatly augmented in both human and murine M1-polarized macrophages relative to both non-polarized and M2-polarized macrophages. It is plausible that macrophage activation by bacterially-derived products could function during colitis, at least in part, to promote the up-regulation of STING protein expression. High levels of STING protein might conceivably sensitize macrophages to limited amounts of cDNs.

That we did not see the complete prevention of DSS colitis in the *Tmem173*^*gt*^ mice was not unexpected given the known participation of various PAMP receptors, as well as the inflammasome, in cells of the innate immune system^2,16^. Also, with regard to the latter, there was an intriguing report that showed that cDNs can stimulate the NLRP3 inflammasome in a STING-independent manner^38^. Thus, in the DSS model, STING-mediated effects are obviously acting in concert with various other pro-inflammatory pathway effectors. However, in view of the multifactorial processes involved in the pathogenesis of colitis, the degree of disease inhibition in the setting of STING deficiency was striking.

While there is clinical data stating that IFN-β is a beneficial therapeutic for the treatment of ulcerative colitis, there are also reports that this therapy in multiple sclerosis or chronic hepatitis patients can worsen disease, or even spontaneously induce IBD^39–41^. In addition, it was reported that the treatment of DSS-treated mice with a *Lactobacillus acidophilus* strain that constitutively expressed IFN-β, increased disease severity^42^. There is also evidence that the loss of the IFNAR1, a critical component of the IFN signaling pathway^43,44^, does not exacerbate or induce colitis^45^. This receptor activates intracellular signal transduction in response to all type I interferons including IFN-β and the various IFN-α subtypes^46^. For example, Tschurtschenthaler *et al*. showed that mice deficient in ifnar1 (in intestinal epithelial cells) did not exhibit spontaneous inflammation or increased severity in DSS colitis compared with ifnar1^+/+^ mice^45^. Finally, a Cochrane database review by Seow *et al*.^47^ found no support for Type I IFN therapy for the induction of remission in patients with ulcerative colitis and recommended that efficacy and safety should be evaluated prior to continuation or initiation of clinical trials.

STING may be involved in homeostatic processes that regulate both inflammation and carcinogenesis^14,15^. For example, chronic STING activation may alter the predisposition to carcinogenesis as noted by Xia *et al*. in which they observed that STING signaling is suppressed in a wide variety of cancers^48^. Moreover, Zhu *et al*.^14^ reported that the presence of STING attenuated inflammation-induced colorectal cancer in mice. Superficially, this appeared to contrast with our findings in which STING deficiency was protective during DSS-induced colitis and that treatments with a STING-agonist dramatically exacerbated disease severity. A few possibilities could account for the apparent discrepancy in these findings, such as differences in the microbiota between facilities, the DSS used (eg. source, concentration, lot, etc.), and more likely, that the disease induction models employed were different. While our model employed DSS treatment over 7 days, in the Zhu *et al*. study, mice were injected with azoxymethane (AOM), a DNA damaging agent that also has effects on the gut microbiome^49,50^ prior to the initiation of DSS exposure. Thus, the basal homeostatic state of the gut lining prior to DSS exposure was likely to be different in the two models. To further add to the ambiguity, a recent study by Canesso *et al*. observed that STING was required for intestinal homeostasis and the control of inflammation and that the loss of STING worsened DSS-induced colitis^17^, whereas in contrast, Ahn *et al*. reported that STING-deficient mice are rescued from spontaneous colitis in IL-10 deficient mice^18^. In summary, our studies suggest that STING acts as a pro-inflammatory mediator of DSS-induced colitis, and this is supported by the observation that colitis was dramatically worsened when the mice were co-treated with a STING agonist.

The possibility that human STING responds poorly to bacterially derived cDNs has been raised^26^, however, this study was limited to peripheral blood mononuclear cells (PBMCs) derived from only 2 individuals. The STING expression levels could potentially have been a factor as some variants have low STING protein and reduced *TMEM173* transcript levels which have shown to be unresponsive respond to cDNs^51^. For example, there is evidence that STING alleles have variable responses to bacterially-derived cDNs within the human population^52,53^ and that sizable fractions of the human population are homozygous for the following *STING* allele: R71H-G230A-R293Q. In view of the fact that there are multiple polymorphisms within the STING cDN binding and dimerization domains^52–54^, it would appear premature to conclude that all human STING variants would show impaired responses to bacterially-derived cDNs.

These results support our hypothesis that STING activation plays a role in the development and the degree of inflammation in the DSS model of colitis. Whether colonic epithelial damage allows free cDNs in the luminal fluid to access cells of the immune system, or whether bacterial phagocytosis is required to deliver cDNs or bacterial dsDNA remains an open question. The development of human STING-specific antagonists is an area of active investigation, particularly for the treatment of inflammatory and autoimmune diseases. To this end, screening efforts in the lab of Ablasser *et al*.^55^, recently identified molecules that appear to suppress STING activation. If our findings can be extrapolated to gut inflammation in humans, then the next stage would be to further develop and utilize STING antagonists as therapeutics in an attempt to limit the degree and to promote the resolution of intestinal inflammation. Conversely, given the adverse impact of the murine STING agonist on DSS-induced colitis in mice, and if this phenomenon develops in humans, then caution is warranted as the therapeutic administration of STING agonists could exacerbate existing or underlying inflammatory disease(s).

## Methods

### Mice

These studies were conducted in accordance with the guidelines established by the Canadian Council of Animal Care, with all protocols approved by the Health Sciences Animal Care Committee at the University of Calgary. Male mice (8–10 wks of age) were fed standard laboratory chow, allowed water *ad libitum*, and were housed in a double barrier unit within individual micro-isolator cages (Techniplast) having HEPA filter lids with individual filtered air and water supplies in a room maintained at 22 ± 1 °C, 65–75% humidity, and a 12-hr light/dark cycle. Male C57BL/6J mice were selected for these studies as they are generally considered more susceptible than females to DSS (increased severity, increased hyperplasia)^56^ and are largely the sex selected for these studies. In addition, while there are studies that suggest that Crohn’s disease is more prevalent in females, in ulcerative colitis population-based studies, no significant differences have been shown^57,58^. However, we acknowledge that there could be differences regarding the role of the STING protein and the sex of the mice.

*Tmem173*^*gt*^ C57BL/6J mice (Jackson Laboratories, USA) do not produce IFN-β in response to cDNs or *Listeria monocytogenes* infection^1^. These mutant mice carry a missense mutation in exon 6 of the transmembrane protein 173 (*Tmem173* or *Sting*) gene, which results in an isoleucine-to-asparagine change at amino acid 199, in the C-terminus of the protein. C57BL/6J mice from the same commercial source were used as the wild-type controls.

Homozygous *Tmem173*^*gt*^ and wild-type mice were administered 3% dextran sulfate sodium (DSS) in their drinking water for 7 days. A second group were treated with 5 or 10 mg/kg/day i.p of DMXAA (5,6-dimethylxanthenone-4-acetic acid), a STING agonist that specifically binds to murine but not human STING^26^, concomitant with DSS treatment. Mice were monitored daily to assess water consumption, changes in body weight, stool consistency, and for the presence of gross fecal blood.

### Histology and immunohistochemistry

Colonic samples (3–4 cm distal to the cecum) were fixed in 10% neutral buffered formalin, embedded in paraffin, and then stained with either hematoxylin and eosin (H&E) or periodic acid-Schiff (PAS)(Calgary Laboratory Services, AB, Canada). Damage was assessed using a previously described histological activity index (HAI)^59^. In brief: epithelial damage, 0 = none, 1 = minimal loss of goblet cells (GCs), 2 = extensive loss of GCs, 3 = loss of crypt and extensive loss of GCs, 4 = extensive crypt and GC loss. Infiltration, 0 = none, 1 = crypt base, 2 = muscularis mucosa, 3 = extensive muscularis mucosa and edema, 4 = submucosal. Colonic crypt depth was measured in 4 locations from 6 untreated WT and *TMEM173*^Gt^ mice; n = 24 total measurements plotted. To assess possible differences in goblet cells, the number of goblet cells per 200 um^2^ area were also counted from the untreated WT and *TMEM173*^Gt^ mice; n = 12.

Paraffin sections were also stained with an antibody against Iba-1 (ionized calcium-binding adapter molecule 1) that is expressed by activated macrophages. Sections were blocked (2% normal goat serum) for 2 hrs at room temperature (RT) and then incubated with the anti-Iba-1 (1:200, rabbit polyclonal, Wako #019–19741) in blocking buffer overnight at 4 °C. The sections were then washed and incubated with biotinylated anti-rabbit IgG antibody (1:200) in blocking buffer for 2 hrs at RT, then incubated with Elite ABC reagent Reagent A (1:100) and Reagent B (1:100) for 1 hr each, and then incubated in peroxidase solution.

### Immunoblotting

For STING protein expression, colonic homogenates (10% w/v), or macrophage lysates, were prepared in extraction buffer (0.15 M NaCl, 5 mM EDTA, 1% Triton-X 100, 10 mM Tris-HCl, pH 7.4) with the addition of a protease inhibitor cocktail (Complete, Roche Diagnostic GmbH). Protein concentrations were determined by Bradford assay. Proteins (50 μg) were separated on 12% SDS-polyacrylamide gels and transferred onto PVDF membranes. Membranes were blocked in 5% BSA/TBST and then incubated with an anti-STING rabbit polyclonal antibody (1:2500 dilution, Cell Signaling Technology Inc.) for 24 hr at 4 °C. An HRP-conjugated donkey anti-rabbit secondary antibody was used. Membranes were then stripped and re-probed with an anti-β-actin antibody as a loading control (Sigma-Aldrich, USA). STING protein bands were visualized with SuperSignal® West Pico chemiluminescence substrate and then the signal density was quantified using Image J (1.52K National Institute of Health, USA). Semi-quantitative analyses were performed by measuring the relative abundance of the murine or human STING protein which was then normalized to β-actin. The background was subtracted from all densitometric measurements.

### Macrophage isolation and polarization

Using aseptic technique, femurs were harvested, and the bone marrow flushed from C57BL/6 and *Tmem173*^*gt*^ mice for macrophage polarization studies. The BMDMs were expanded in culture for 7 d in the presence of L-929 mouse fibroblast conditioned medium as a source of M-CSF. Cells were re-seeded onto 6-well plates at 2 × 10^6^ cells/well and polarized for 48 hrs with the addition of 50 ng/ml LPS (List Biological Labs) and 50 ng/ml murine IFNγ (Cedarlane) for M1, 40 ng/ml murine IL-4 (R&D Systems) for M2 or left untreated for M0 cells. These cells were subsequently treated with either 20 μg/ml DMXAA (Cedarlane), 20 or 40 μg/ml 2′3′-cGAMP or 20 μg/ml c-di-AMP (InvivoGen, San Diego, CA) for an additional 6 hrs in the absence of polarizing cytokines. cDN treatments were given in the presence of Lipofectamine-2000 (LF2000, Invitrogen) to allow for entry across the cell membrane^11^. BMDM cultures were all maintained at 37 °C in a 5% CO_2_ humidified incubator.

### Differentiated THP-1 monocyte polarization

We also differentiated human THP-1 monocytes into macrophages with low dose (16 nM) phorbol 12-myristate 13-acetate (PMA) for 72 hr, followed by a 48 hr resting period (washed out with media: RPMI + 5% FCS). To obtain an M1 phenotype, the macrophages were polarized for 48 hr with media containing 50 ng/mL human IFNγ (Cedarlane) and 50 ng/mL LPS; for the M2 phenotype, cells were incubated with 40 ng/mL human IL-4 (Cedarlane) for 48 hr.

### Real time RT-PCR

Real-time RT-PCR was used to examine transcriptional changes of several cytokines as well as markers of M1 or M2 polarized macrophages. These genes included *IFN-β*, *CXCL10*, *iNOS*, *Arg-1*, *Fizz1* and *IL-12p40*. Colonic tissues from all groups of mice were placed in QIAzol reagent (Qiagen), homogenized, and the total cellular RNA was extracted according to the manufacturer’s instructions. First-strand cDNA was synthesized from 1 μg total RNA in a 25-μL reaction volume and then treated with DNAse (Promega) followed by RT-PCR with 10 mM dNTPs, random primers (Roche) and Superscript II reverse transcriptase (Invitrogen). Duplicate independent quantitative real-time PCR was performed using the LightCycler System (Roche Diagnostics GmbH). SYBR Green I (Roche Diagnostics GmbH) was used to visualize and monitor the amplified product in real time. Gene-specific oligonucleotide primers were designed for the genes of interest. β-Actin was amplified as an internal control. The change in fluorescence of SYBR Green I dye in each cycle was determined using the LightCycler system software (Roche Diagnostics GmbH), and the threshold cycle above background for each reaction was calculated. Data was normalized to β-actin mRNA and experimental transcripts expressed as the relative fold change in mRNA. Primer sequences for *IFN-β*, *CXCL10*, *iNOS*, *Arg-1*, *Fizz1* and *IL-12p40* were as previously described^11^.

### Statistical methods

Results were expressed as mean ± SEM. For the *in vivo* studies, comparisons amongst groups of data were made using a one-way analysis of variance (ANOVA) followed by a *post-hoc* Tukey’s test. For the macrophage studies, samples were compared via 2-way ANOVA with Bonferroni’s *post-hoc* test. A *P*-value of less than 5% was considered significant.
